# Cholestatic jaundice, acute kidney injury and acute pancreatitis secondary to the recreational use of methandrostenolone: a case report

**DOI:** 10.1186/1752-1947-5-138

**Published:** 2011-04-06

**Authors:** Greg A Rosenfeld, Albert Chang, Michael Poulin, Peter Kwan, Eric Yoshida

**Affiliations:** 1 Department of Medicine, University of British Columbia, 5th Floor, Gordon and Leslie Diamond Health Care Centre, 2775 Laurel St, Vancouver, BC, V5Z 1M9, Canada; 2 Department of Pathology, Vancouver General Hospital, 889 West 12th Avenue, Vancouver, BC, V5Z 1M9, Canada

## Abstract

**Introduction:**

Over the last few years the use of anabolic steroids has become increasingly common amongst amateur athletes and for aesthetic purposes. As a result, the adverse events related to their use are being seen more frequently. Methandrostenolone is an anabolic steroid which is widely available and has been used for both performance enhancement and aesthetic purposes. This drug has also been reported to cause cholestasis of the intra-hepatic bile ducts resulting in elevated aminotransferases, hyperbilirubinemia and clinical jaundice. However, to the best of our knowledge this agent has not been previously reported to cause pancreatitis or acute kidney injury.

**Case presentation:**

In this paper, we report the case of a 50-year-old man of Indian descent who presented with a six week history of diffuse abdominal pain, anorexia and weight loss following an eight week cycle of methandrostenolone use. At initial presentation, his lipase level was 785 U/L, bilirubin was 922 μmol/L and creatinine was 200 U/L while his aspartate aminotransferase and alanine aminotransferase levels were only mildly elevated at 61 U/L and 56 U/L respectively. His lipase peaked on day nine at >3000 U/L whilst his creatinine level was 299 U/L. Imaging was consistent with acute pancreatitis while a liver biopsy was consistent with intra-hepatic cholestasis and a kidney biopsy revealed evidence of acute tubular necrosis.

**Conclusion:**

Both acute pancreatitis and acute kidney injury have rarely been reported with anabolic steroid use and they have not been previously reported to occur in the same patient. This case demonstrates some potentially new and serious adverse consequences occurring with the use of anabolic steroids, of which physicians need to be aware.

## Introduction

Anabolic androgenic steroids (AAS) have been in widespread use amongst elite athletes to enhance performance for decades [1]. Major League Baseball and the National Football League have provided numerous examples of steroid use amongst their professional athletes. Several Olympic athletes have tested positive for the use of AAS or admitted to their use [2]. Meanwhile, the Vancouver 2010 Winter Olympic games saw the creation of the most sophisticated anti-doping testing laboratory to date, resulting in 30 athletes testing positive and being banned from attending the games prior to their opening. With the knowledge of widespread steroid use has come an increased awareness of the adverse effects and sometimes serious consequences of AAS use. Nevertheless, there seems to be an ever increasing use of these agents by recreational athletes and for aesthetic purposes. Recent estimates place AAS use in the USA and Sweden at 1% of the population and we can reasonably assume that the rates of use in Canada are similar [1]. The internet has increased the black market availability of these drugs without prescription and consumers are frequently unaware of the risks of taking these drugs.

Methandrostenolone (Dianabol) was first introduced as an anabolic steroid by Ciba in the 1960s. Methandrostenolone was one of the AAS used to enhance athletic performance by the former East German Olympic program [3]. This agent has numerous side effects common to anabolic androgenic steroids which include gynecomastia, acne, mood changes (aggressiveness) and testicular atrophy [4]. Stanozolol, another carbon-17-alkylated anabolic steroid, has been previously reported to cause severe cholestasis and acute renal failure in a young athlete [5]. Acute kidney injury arising from the use of anabolic steroids and vitamin supplementation in two male athletes has also been recently reported [6].

Methandrostenolone has also been reported to cause cholestasis of the intra-hepatic bile ducts resulting in elevated aminotransferases, hyperbilirubinemia and clinical jaundice [7]. In this paper, we present a case of pancreatitis, cholestasis of the liver and acute kidney injury associated with the use of methandrostenolone for aesthetic purposes in a 50-year-old, non-athlete man. A brief literature review of Medline and PubMed Central, utilizing the search terms 'androgenic anabolic steroids', 'pancreatitis' and 'methandrostenolone', failed to reveal any other cases of pancreatitis arising from the use of anabolic steroids.

## Case presentation

A 50-year-old man of Indian descent, known to have mild, chronic hepatitis C, presented with a two week history of diffuse abdominal pain. Six weeks prior to the onset of the pain, he had a gradual onset of anorexia and a 20 pound weight loss. Our patient noticed darkly coloured urine and pale stools beginning around the time of the onset of pain. He had not received treatment for his hepatitis C infection. He had intermittent and occasionally heavy alcohol consumption on weekends. He had also been taking methandrostenolone: 10 mg orally twice a day, five days a week for three weeks and then three times a day, five days a week for the next five weeks, for a total of eight weeks immediately prior to presentation.

When he presented, his white blood cell count was 9.8 giga/L (normal range 4.0-11.0 giga/L), his hemoglobin level was 172 g/L (normal range 135-170 g/L) and his platelet levels were 378 giga/L (normal range 150-400 giga/L). His lipase level was 785 U/L (normal range 0-393 U/L), gamma-glutamyltransferase (GGT) level 24 U/L (normal range 15-80 U/L), alkaline phosphatase (ALP) level 154 U/L (normal range 50-160 U'L), total bilirubin level 922 μmol/L (normal range 0-18 μmol/L), with direct bilirubin 804 μmol/L (normal range 0-5 μmol/L), alanine aminotransferase level (ALT) 56 U/L (normal range 25-80 U/L), aspartate aminotransferase (AST) level 61 U/L (normal range 10-38 U/L) and lactate dehydrogenase level 242 U/L (normal range 90-210 U/L). His international normalized ratio was 1.1 and serum albumin level was 35 g/L (normal range 34-50 g/L). He was also noted to have an element of acute renal failure with a serum creatinine level of 200 μmol/L (normal range 60-115 μmol/L) (Table 1). He had no previous history of renal disease. He was admitted to our hospital for supportive management and further investigations.

**Table 1:** Table showing laboratory values over time in hospital and in the first few weeks post discharge.

	WBC	HGB	BUN	CR	AMYLASE	LIPASE	GGT	ALP	BILI	ALT	AST
Admission	8.8	172	13.2	200		785	24	154	922	56	61
Post admission Day 1	12.5	148	11.9	175		634	13	133	755	45	51
Day 2		158	10.9	171			38	161	869	45	53
Day 3	10.8	160	11.3	185	71	968	10	135	739	33	42
Day 4	11.5	164	13.8	199	29	345	24	172	919	46	72
Day 5			16.7	290	47	417	18	182	937	48	67
Day 6	10.9	153	19.7	288	50	461	14	180	896	52	67
Day 7	13.9	170	24.5	298	141	1629	24	206	804	56	70
Day 8	16	161	24.3	291	1855	>3000	16	165	678	44	73
Day 9	14.1	136	26.4	299	1720	>3000	8	171	660	64	96
Day 10	17.1	137	22	246	841	>3000	8	151	544	47	54
Day 11	17.2	127	15	206	343	>3000	9	161	532	44	48
Day 12	15.8	121	12.3	179			9	159	473	35	45
Day 13	16.2	120	9.6	161			9	146	447	28	36
Day 14	18.7	123	9.4	153			9	119	424	27	38
Day 15	17.3	109	9	160			13	145	409	33	44
Day 16	20.7	111	7.9	136			13	122	302	24	31
Day 17				140	68	412	11	136	247	29	37
Day 18	18.6	112	5	120			14	1483	166	32	37
Discharge (Day 19)	13.5	102	5.1	131			13	130	136	33	37
At follow up (Day 38)	11.8	131	4	119	64	237	23	145	53	92	59
(Day 39)	10.5	125	3.9	103	46	170	17	128	50	84	67

A non-contrast computed tomography (CT) scan of his abdomen was performed shortly after admission which showed mild fatty infiltration of the liver. There was no evidence of inflammatory fat stranding around his pancreas or kidneys. An ultrasound of his abdomen performed 48 hours later showed mild hepatic enlargement with his liver measuring 17.1 cm in length with a coarse, echogenic texture. There were no focal hepatic lesions or intra-hepatic duct dilatation. There was a small amount of sludge in his gallbladder but no stones. His common bile duct was of normal caliber at 2 mm in diameter. His pancreas was well seen and unremarkable. His renal parenchyma was echogenic and measured at the upper limits of normal size which was in keeping with medical renal disease.

Within a couple of days of admission, our patient began to experience worsening nausea, vomiting and abdominal pain. His serum lipase level declined over the first three days, but it later began to rise and peaked on day nine at > 3000 U/L, while his ALP level also peaked on day eight at 206 U/L. His ALT and AST levels remained only mildly elevated. Our patient's creatinine level rose to a peak of 299 μmol/L on day nine.

Serum auto-antibodies, serum protein electrophoresis and cryoglobulins were all negative. Human immunodeficiency virus antibodies were negative however, as anticipated, his hepatitis C viral RNA was qualitatively positive. His fasting serum lipid levels were low with the exception of triglycerides which were mildly elevated at 3.25 mmol/L (normal range 0.60-2.30 mmol/L). Our patient's clinical picture was most consistent with acute pancreatitis and thus, a non-contrast (due to renal failure) CT scan of his abdomen was obtained on the tenth day. This showed a bulky pancreas with adjacent inflammatory fat stranding which was interpreted as consistent with pancreatitis without a focal drainable abscess. There were also no signs of chronic pancreatitis.

**Figure 1:**
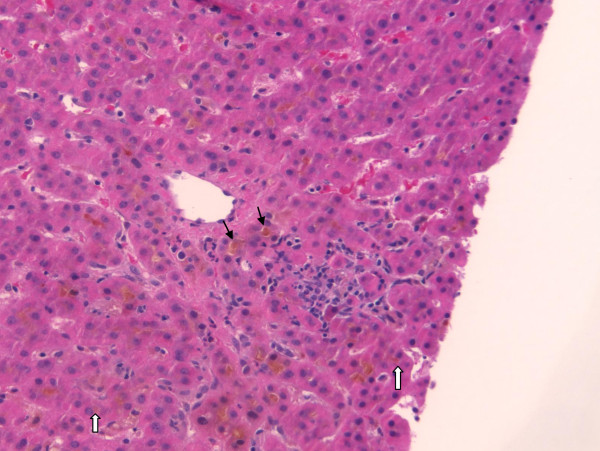
**Liver cholestasis due to methandrostenolone**. Core liver biopsy showing bile filled canaliculi (black arrows) and bile in the hepatocytes (white arrows). As well, there are plasma cells and periportal inflammation.

Our patient went on to have a liver biopsy which revealed grade 2 portal and lobular inflammation, stage 2-3 fibrosis consistent with hepatitis C viral infection, moderately severe acute cholestasis consistent with anabolic steroid use, and mild pericellular fibrosis consistent with alcohol abuse but without evidence of steatosis or steatohepatitis (Figure 1). A renal biopsy was also performed which showed acute tubular injury of uncertain etiology. His glomeruli were normal and there was evidence of desquamation of his tubular epithelial cells. Therefore, acute tubular necrosis was confirmed as the etiology of his renal failure.

With supportive therapy, our patient's pancreatitis began to resolve and he was asymptomatic at the time of discharge. One month after discharge, his renal function had returned to normal and his amylase (46 U/L), lipase (270 U/L), GGT (17 U/L), and ALP (128 U/L) levels had all returned to normal. His total bilirubin level remained mildly elevated at 50 μmol/L and his ALT (84 U/L) and AST (67 U/L) levels were also mildly elevated in keeping with his chronic hepatitis C infection.

## Discussion

AAS use or misuse is no longer solely by elite athletes seeking enhanced performance. Teenage boys in Sweden reported using AAS for a variety of reasons [8], while bodybuilders, weight-lifters and prison populations have also been shown to have higher levels of misuse [9]. Our patient took AAS not for athletic performance but for aesthetic reasons. He reported wanting to "remain in shape" as the main reason for taking these pills. Anabolic steroids are readily available to the general public over the internet and at public gyms, they are easily obtained illegally, without a prescription. As a result, patients are less likely to report taking AAS to their physician and physicians are less likely to consider the possibility of the use of AAS in the non-athlete population.

Our patient represents the first case of a patient developing pancreatitis as a result of anabolic androgenic steroid use. A recent clinical workshop reviewed the criteria necessary for reporting cases of Drug Induced Liver Injury [10]. We believe that this case report meets those criteria and we have reported on all of the necessary elements and many of the supportive elements outlined in the summary document emanating from that workshop. The usual causes of pancreatitis were excluded in our patient. He did not have any evidence of gallstones on repeated imaging studies, nor were his alkaline phosphatase or GGT significantly elevated as would be expected with obstructing gallstones. His triglyceride levels were only mildly elevated and not to the degree usually seen with pancreatitis. He had been hospitalized for four days before the first sign of pancreatitis and had not consumed alcohol for at least a week at that time. Given that his liver biopsy confirmed a cholestatic picture consistent with steroid use, we conclude that this was the main contributing factor in the pathogenesis of his pancreatitis. His hepatitis C may have made him more prone to liver injury from steroid use but the relatively low elevation of transaminases suggests that his hepatitis C infection was quite mild and stable. Furthermore, his liver biopsy showed a cholestatic pattern much more in keeping with steroid use than hepatitis C infection.

Our patient's acute kidney injury is unique in that the pathology showed acute tubular necrosis (ATN). In two previous case reports with anabolic steroid use and vitamin supplementation as the cause of acute kidney injury, the biopsies showed acute interstitial nephritis [6]. In these cases, the kidney injury was attributed to the supplementation of vitamin D with resultant hypercalcemia as the mechanism of kidney injury. Excessive vitamin intake and hypercalcemia were not factors in our patient. The extremely high bilirubin level found in our patient is consistent with a previously reported case of severe cholestasis and ATN secondary to AAS use [5]. In that report, the proposed mechanism of ATN was secondary to severe cholestasis and the increased renal excretion of bilirubin. Two additional cases of acute kidney injury associated with the use of an over-the-counter nutritional supplement (Superdrol™) have been reported [11,12]. In the first case a kidney biopsy was not performed, while the second case reported a biopsy consistent with IgA nephropathy [12]. Whatever the exact mechanism, our patient's history is most consistent with his AAS use as the main culprit in both his acute kidney injury and pancreatitis.

## Conclusion

AAS use and misuse is being seen in an expanding population of patients because they are readily available and often perceived as safe. The side effects and risks of taking AAS are difficult to assess in controlled trials due to the unethical nature of administering these drugs in the doses usually taken by patients who use them for aesthetic or athletic purposes. As a result, with the increasing use of these drugs, we can expect to see an increase in previously unreported adverse consequences. Although there have been previous reports of severe cholestasis and jaundice with the recreational use of anabolic steroids [13], this is the first case report where acute pancreatitis and acute kidney injury also resulted from such recreational use. Physicians need to be aware of the risks to their patients who consume AAS, and to consider that even with only mild elevations of aminotransferases, serious consequences such as cholestasis, acute kidney injury and pancreatitis may result.

## Consent

Written informed consent was obtained from the patient for publication of this case report and any accompanying images. A copy of the written consent is available for review by the Editor-in-Chief of this journal.

## Competing interests

The authors declare that they have no competing interests.

## Authors' contributions

GR and AC were major contributors in writing the manuscript. EY and PK analyzed and interpreted the patient data regarding the patient's presentation and provided the clinical care of the patient. MP performed the histological examination of the liver biopsy and prepared the figure for the manuscript. All authors contributed to the writing of the manuscript and approved the final version.
